# Electric Field Oriented Nanostructured Organic Thin Films with Polarized Luminescence

**DOI:** 10.1186/s11671-017-1936-9

**Published:** 2017-03-04

**Authors:** I. D. Karbovnyk, I. Olenych, I. N. Kukhta, A. Lugovskii, G. Sasnouski, T. Chutora, A. P. Luchechko, I. Khalakhan, A. Kukhta

**Affiliations:** 1 Department of Electronics and Computer Technologies, Ivan Franko National University of Lviv, Lviv, Ukraine; 2 Institute of Chemistry of New Materials, National Academy of Sciences of Belarus, Minsk, Belarus; 3 Institute for Applied Physical Problems, Belarusian State University, Minsk, Belarus; 4 Regional Centre of Advanced Technologies and Materials, Faculty of Science, Joint Laboratory of Optics of Palacký University and Institute of Physics AS CR, Olomouc, Czech Republic; 5 Department of Surface and Plasma Science, Faculty of Mathematics and Physics, Charles University, Prague, Czech Republic; 6 Institute for Nuclear Problems, Belarusian State University, Minsk, Belarus; 7 Tomsk State University, Tomsk, Russia

**Keywords:** Luminescence, Liquid crystalline molecules, Thin films, Morphology

## Abstract

The effect of the external electric field of 10^5^ V/m on the ordering of two luminescent liquid crystalline molecules (1-pentyl-2^/^,3^/^-difluoro-3^///^-methyl-4^////^-octyl-*p*-quinguephenyl and 9,10-Bis (4-pentylphenylethynyl)antracene) during thermal vacuum deposition is studied. The morphology, electrical conductivity, optical absorption, luminescence spectra, and polarization are presented and analyzed. All data show the formation of ordered films. The polarization degree is 60% for 1-pentyl-2^/^,3^/^-difluoro-3^///^-methyl-4^////^-octyl-*p*-quinguephenyl oriented films and 28% for 9,10-Bis (4-pentylphenylethynyl)antracene. The lower value of M2 luminescence polarization can be explained by the absence of dipole moment in this molecule.

## Background

Orientation of π-conjugated molecules across a large area without defects is attractive for development of manifold electronic devices. Commonly, self-organization of molecules is the subject of elevated interest [1]. Luminescent thin films with ordered molecules can be very interesting as active parts of organic electroluminescent (EL) diodes emitting polarized light without any polarizers resulting in efficiency decrease. In principle, there is a variety of methods to align organic EL materials for polarized emission. Oriented Langmuir-Blodgett films [2], mechanically aligned films, e.g., stretched films [3] or films using direct rubbing procedures [4], substrate induced ordered films [5], and epitaxial vapor deposited films, [6] are widely known. Authors [7] showed the presence of self-organization of molecules in electric field due to induced electric dipole in molecules. Liquid crystalline (LC) molecules are known to align easily in electric field. Fluorescent LC molecules are very attractive nowadays [8]. Polymeric LC molecules are already used in polarized EL devices [8]. However, they require an additional orienting layer.

In this study, we fabricated a polarized fluorescent thin film by means of the vapor deposition of fluorescent liquid crystalline molecules in the presence of an electric field. Optical, luminescent, morphological, and electrical properties of these films are presented.

## Methods

### Sample Preparation

**Fig. 1:**
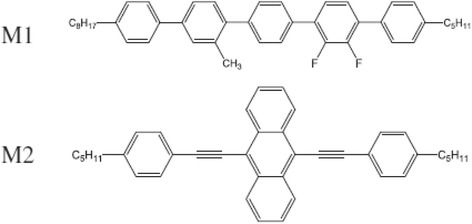
Chemical structures of M1 and M2

1-pentyl-2^/^,3^/^-difluoro-3^///^-methyl-4^////^-octyl-*p*-quinguephenyl (M1) and 9,10-Bis (4-pentylphenylethynyl)antracene (M2) organic molecules were used for experimental studies. Both molecules are highly luminescent liquid crystalline compounds capable to be deposited by thermal evaporation from solid and by spin coating from solutions. Both molecules were obtained and purified as described earlier [9]. Chemical structure of M1 and M2 is shown in Fig. 1.

M1 and M2 films were thermally evaporated and deposited in 10^−4^ mmHg vacuum using VUP-5 M machine. This deposition technique and instrumentation was earlier used to fabricate nanostructured metallic films with random structure [10–12] and amorphous SiO_*x*_ films that passivate luminescent silicon nanocrystals [13].

Optical glass with transparent and electrically conductive SnO_2_ coating layer was used as a substrate material for oriented film deposition. The conductive layer was required in order to apply an electric field during the deposition process. Before the deposition, all substrates were carefully cleaned with ethanol. The thermal stability of M1 and M2 under evaporation is known to be high [14]; therefore, both compounds are capable to be deposited by thermal evaporation from solid.

**Fig. 2:**
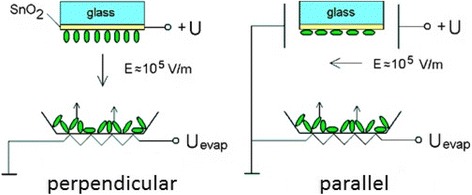
Schemes of electric field application for controlling the spatial orientation of organic molecules perpendicularly and in parallel to the surface

Spatial orientation of organic molecules during thermal deposition was performed by applying an electric field in different geometries. The technique is explained in Fig. 2.

In the first geometry (Fig. 2, left), the voltage was applied to SnO_2_ layer on top of the glass substrate which was fixed at the distance of 6 cm from the tungsten evaporator. Resulting electric field was perpendicular to the substrate surface. In this configuration (Fig. 2, right), the substrate was placed between electrodes and aligned in parallel with the electric field vector. In both cases, the field was about 10^5^ V/m. During the evaporation process, organic molecules M1 and M2 are expected to align along the electric field lines, eventually forming oriented layers on the substrate. The substrate was kept at chamber temperature of about 20 °C. The average rate of deposition was about 10 nm per second, which is relatively high. It was slightly different for M1 and M2. Average thickness of deposited films was interferometrically measured to be in the range of 100–150 nm for both molecules.

### Structural, Electrical, and Optical Characterization

Topology of obtained organic films was characterized by atomic force microscopy (AFM) [15]. The analysis was performed using a Bruker MultiMode 8 microscope under ambient environmental conditions. The height images were acquired in tapping mode using silicon cantilevers (Bruker SCANASYST-AIR).

Dark current-voltage curves were recorded at room temperature at ambient conditions according to standard technique [16] using a V7-21 digital voltmeter and stabilized voltage source. Current flow through the film volume was normal to the film substrate. SnO_2_ layer on the glass substrate was used as the first electrode. The sheet resistance of this layer was 20 Ω/sq. Spring round-shaped silver contact (~2 mm in diameter) was used as the second electrode.

Optical absorption spectra, photoluminescence spectra, and photoluminescence excitation spectra of the oriented organic films were studied using a CM2203 spectrofluorometer (Solar, Belarus). The range of spectral measurements was 270–700 nm, and experiments were performed at room temperature.

### Molecular Structure Simulation

The equilibrium geometry of the ground electronic state and dipole moments of M1 and M2 free molecules were calculated by the method of functional density theory (DFT) using the B3LYP hybrid exchange-correlation functional [17, 18] and the 6-31G (d, p) basis set in the framework of the Gaussian-09 program package [19].

## Results and Discussion

**Fig. 3:**
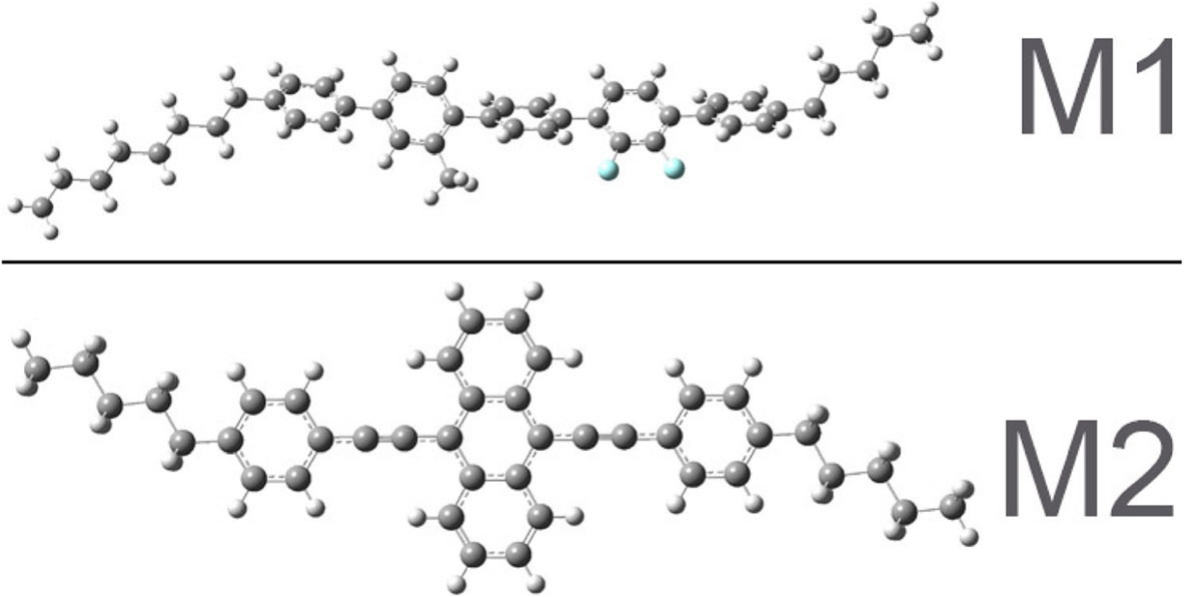
Equilibrium geometries of M1 and M2 molecules

In order to better understand the behavior of these molecules, we calculated their equilibrium geometries in the ground electronic state. The results of these calculations are presented in Fig. 3.

It can be seen that both molecules are really linear, but their aliphatic tails are essentially skewed from the molecular axes. M2 is practically flat, but phenyl rings of M1 are rotated with respect to each other and this molecule is not flat. Non-planar structure and methyl group can essentially prevent M1 molecules from aggregation. We have also calculated dipole moments of these molecules. Dipole moment of M1 was found to be 2.17 D, while dipole moment of M2 molecule is about zero. As a result, different effects of electric field can be expected. M1 molecules are considerably larger and have large dipole moment; therefore, they can be much more sensitive to the direction of the applied electric field than M2. Also, calculation showed that interaction between molecules does not have essential influence on the molecular ordering, though non-covalent interactions can strongly affect molecule packing as well as optical and electrical properties [20].

**Fig. 4:**
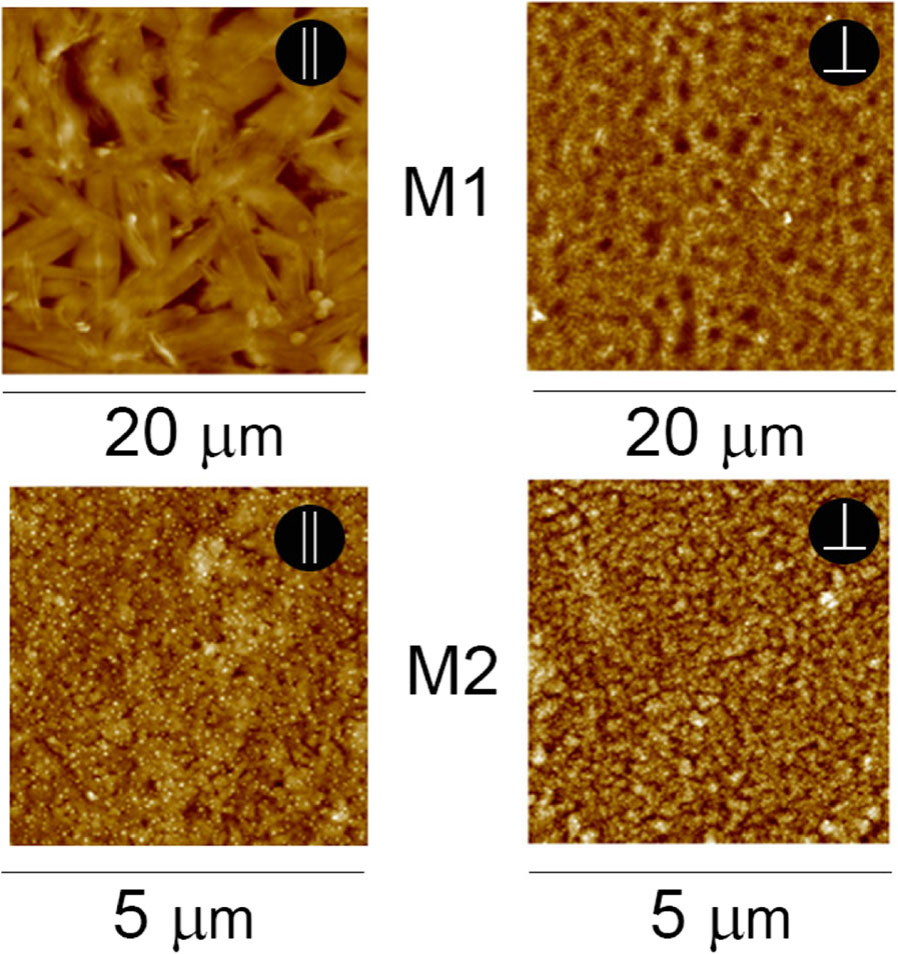
Morphology of M1 and M2 organic film surfaces with different orientation of layer-forming molecules as seen by AFM

Figure 4 shows the AFM images of M1 and M2 organic films. There is a clear indication of prevailing orientation of M1 molecules for different directions of the applied field (see upper part of Fig. 4). It can be seen that for parallel direction M1 molecules form clusters along the substrate surface, and for perpendicular direction, they are located perpendicularly to the surface. It is not clear how molecules are located in these clusters, but within the cluster, molecules have apparently predominant direction. This effect of electric field is much less pronounced in the case of M2. Independently, on the conditions of film deposition, we can see two types of clusters such as needle-like and volumetric. When the applied field is perpendicular to the substrate, volumetric clusters are essentially bigger. The observed difference in electric field behavior of these molecules can be explained by the difference in the dipole moment. More specific techniques like positron annihilation spectroscopy may be needed to analyze the details [21, 22], but it can be noticed that the morphology of vacuum deposited organic films depends strongly on the type of substrate and its temperature [23].

**Fig. 5:**
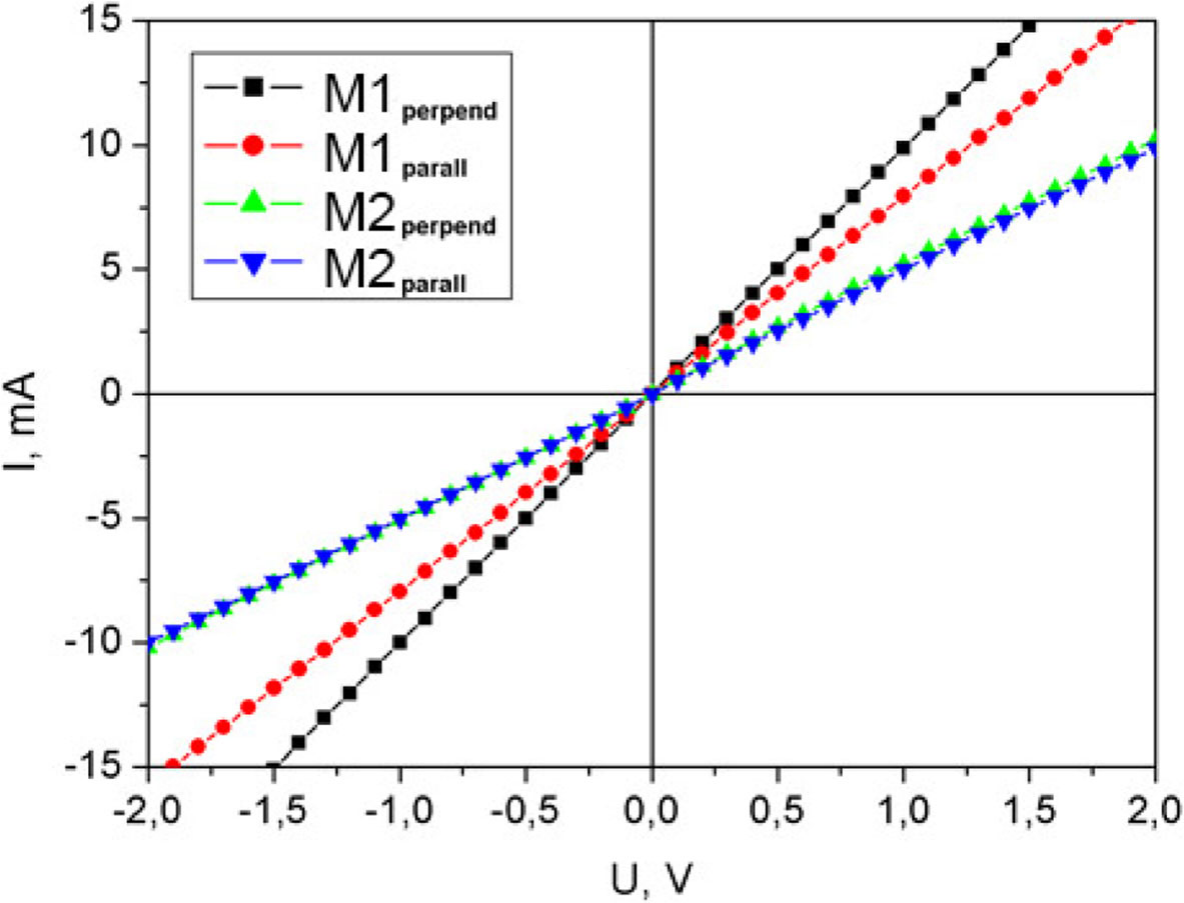
Current-voltage characteristics

All organic film structures under study exhibit linear current-voltage characteristics (see Fig. 5) indicating no energy barrier at the contact-film boundaries.

DC conductivity is higher for M1 films with respect to M2 films. M1 film conductivity was shown to be dependent on film orientation (for M2 films, this effect is negligible). In terms of resistance, for M1 films with molecules oriented perpendicularly to the surface, measured film resistivity was 100 Ω while for M1 films with molecules oriented along the surface, plane film resistivity was about 125 Ω. Such anisotropy in resistance might be related with charge transfer along the molecular chain. Current difference can be observed in the samples where ordered molecules are located perpendicularly to the charge flow and in parallel to the charge flow. M2 film shows approximately the same resistance (200 Ω) for both orientations. It should be noted that the thickness of the films with different orientation was practically the same. The absence of conductivity dependence on electric field direction for M2 films may indicate the weaker effect of the field on the molecular anisotropy in this case.

**Fig. 6:**
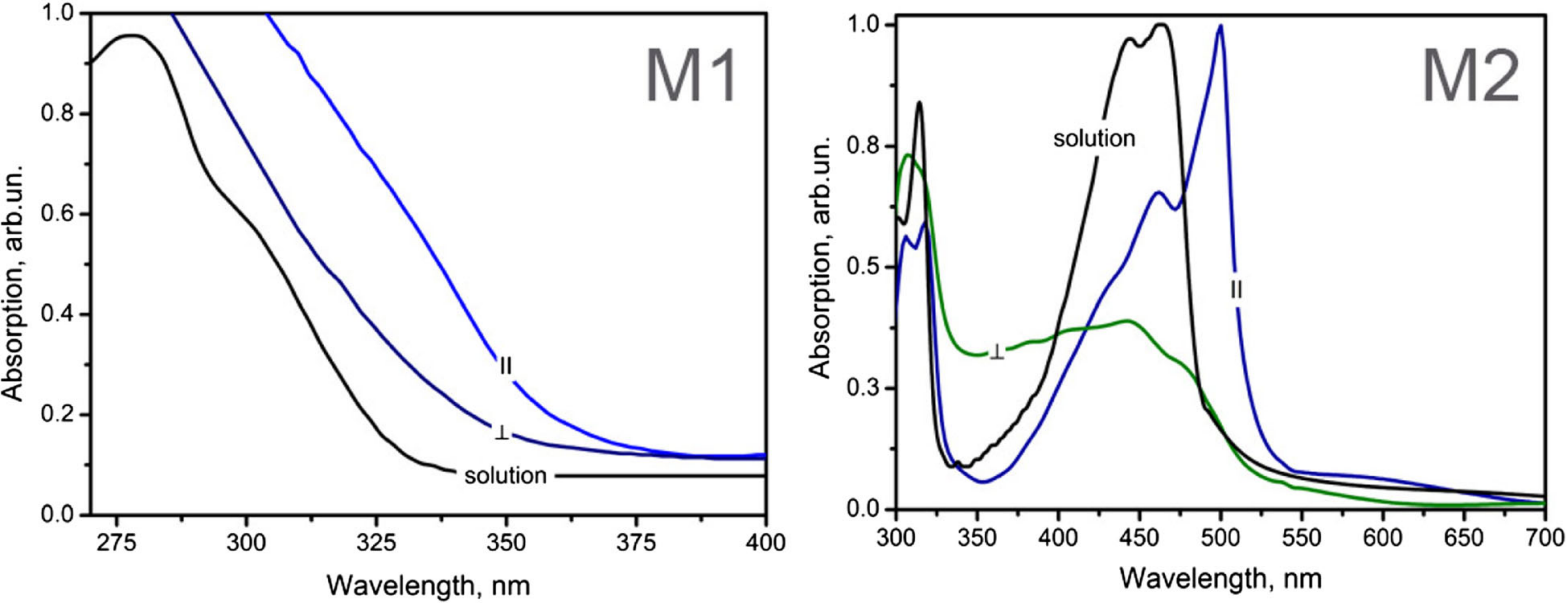
Absorption spectra of M1 and M2 deposited on SnO_2_-coated glass with and without applying electric field. Absorption spectra of M1 and M2 chloroform solutions are shown for comparison

Figure 6 shows optical absorption spectra of M1 and M2 organic films with different prevailing orientation of molecules. Absorption spectra of diluted (less than 10^−6^ M/l) chloroform (CHCl_3_) solution of both substances are given for comparison. Spectral range of 270–400 nm is emphasized in the left part of Fig. 6 as the most crucial changes of the absorption coefficient for M1 occurs in this region. In M1 solution, two distinguished bands at 280 and 303 nm are clearly observed.

In the left part of Fig. 6, one can observe a noticeable absorption band shift which is a characteristic for transitions from liquid to solid phase. Resolution of individual bands in case of M1, however, is somewhat complicated as the SnO_2_ layer is strongly absorbing below 310 nm. The long-wave absorption band is essentially higher for the parallel oriented film corresponding with molecular absorption nature of transversal light wave.

The spectrum of M2 film (Fig. 6, right) with assumed parallel prevailing orientation is shifted to longer wavelengths. M2 film deposited with applied electric field perpendicular to the substrate exhibits some differences in absorption spectrum: sharp peak at ~500 nm diminishes while broad absorption is observed between 350–450 nm. Such behavior may result from the fact that aggregates of molecules with a wide size distribution are formed in this geometry due to π-π interaction between ordered molecules. Actually, the increased aggregate formation in phthalocyanine molecules in electric field has been observed [7].

**Fig. 7:**
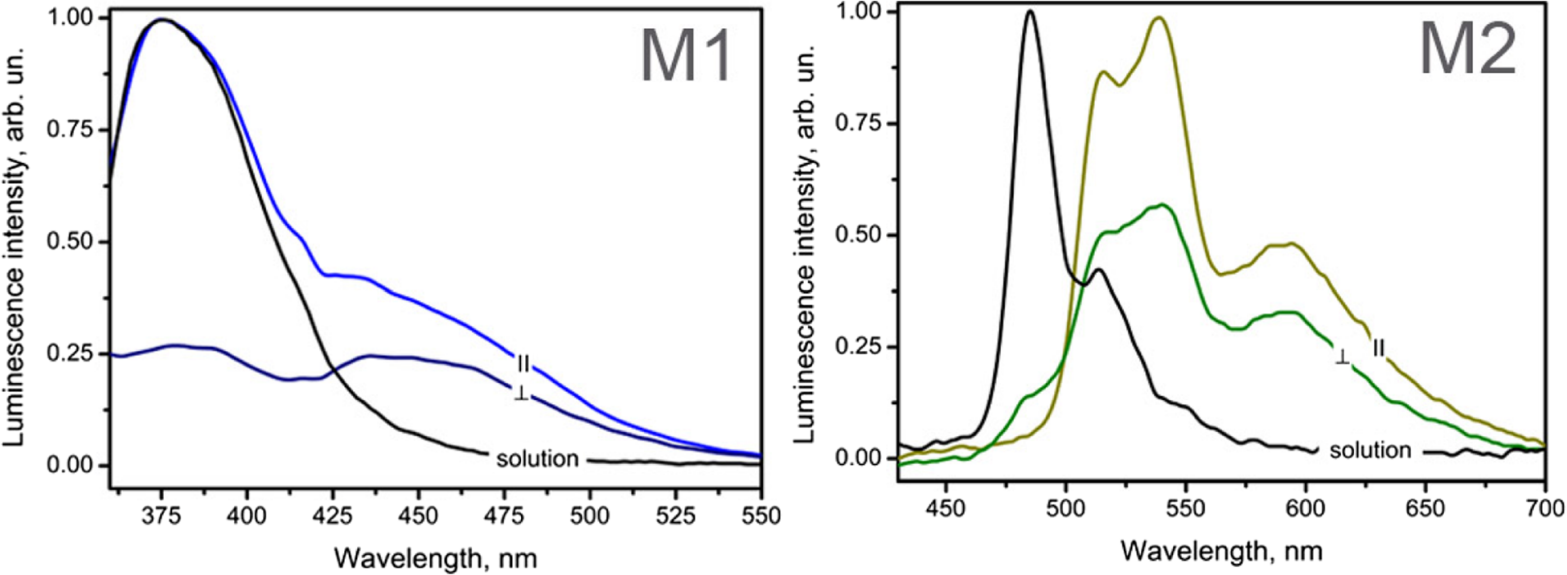
Photoluminescence spectra of M1 and M2 deposited on SnO_2_-coated glass with parallel and perpendicular applied electric field. Spectra of M1 and M2 chloroform solutions are shown for comparison

In Fig. 7, photoluminescence spectra of M1 and M2 solutions and M1 and M2 films are shown. Luminescence of M1 solution is characterized with a single blue emission band corresponding to π-π transition at ~375 nm. For both perpendicular and parallel oriented M1 films, the 375 nm π-π transition band and another less intensive ~440 nm band owing possibly to molecular aggregation. Luminescence yield is generally higher for the M1 film with parallel prevailing orientation.

For M2 solution and films, we also observed spectra of π-π transition (see Fig. 7, right). Additional feature at ~600 nm can be considered as molecular aggregation.

The difference between luminescence intensity in both cases can be attributed to different distribution of molecules in these films. Based on the photoluminescence measurements, we calculated the degree of linear polarization *ρ* by using the equation [24] *ρ* = (*I*_ǁ_ − *I*_⊥_)/(*I*_ǁ_ + *I*_⊥_), where *I*_⊥_ and *I*_||_ are the fluorescent intensities of perpendicular and parallel components, respectively. The polarization degree for M1 oriented films is 60% and 28% for M2. The lower value of M2 luminescence polarization can be explained by the absence of dipole moment in this molecule. In order to obtain more reliable polarization data, we also used polarized light for excitation of these films and registered polarization components with thin film polarizer. In this case, the thickness of the film and exciting light intensity is practically the same. The incident light entered the film at 30° and registered at 60°. We obtained the average polarization of 45% and 70% for M1 and M2 films. Though polarization depends on the geometry of measurements, the obtained results confirm the formation of ordered morphology.

## Conclusions

Morphological studies show stronger orientation of M1 molecules with respect to M2 at the same level of the applied electric field. Electrical measurements indicate that differently oriented M1 layers have different electrical DC conductivity. Signs of specific molecular aggregation for M2 films deposited with electric field applied perpendicularly to the substrate are observed by AFM. This effect has an influence on the optical absorption spectra of M2 oriented film. Comparison of luminescence intensity allowed to estimate the polarization degree for M1 molecule in an electric field reaching 60% for M1 oriented films and 28% for M2. The difference in polarization degree can be explained by the difference in dipole moments.

## Abbreviations

AFM: Atomic force microscopy
DC: Direct current
DFT: Density functional theory
EL: Electroluminescent
LC: Liquid crystalline
